# Factors Associated With Depressive Symptoms Among U.S. Older Chinese Immigrants

**DOI:** 10.1093/geroni/igaa057.542

**Published:** 2020-12-16

**Authors:** Ying-Yu Chao, Jin Young Seo, Mei-Lan Chen, Peijia Zha

**Affiliations:** 1 Rutgers School of Nursing, Newark, New Jersey, United States; 2 Hunter College, CUNY, New York, New York, United States; 3 Georgia State University, Atlanta, Georgia, United States; 4 Rutgers, The State University of New Jersey, Newark, New Jersey, United States

## Abstract

Purpose: Chinese Americans represent the largest Asian ethnic subgroup in the United States. Depression is the most common mental health problem among older adults. However, we have a limited understanding of depressive symptoms among older Chinese immigrants. The study aimed to examine the potential factors associated with depressive symptoms among older Chinese immigrants in U.S. Methods: We recruited participants from psychiatric clinics who sought professional help in New York City. Inclusion criteria were Chinese immigrants from Asian countries 50 years or older; able to speak and understand either Mandarin or Cantonese; and had a diagnosis with major depressive disorder. Depressive symptoms were measured with Quick Inventory of Depressive Symptomatology; cognitive function was measured with Montreal cognitive assessment; sleep quality was measured with Pittsburgh Sleep Quality Index, and physical activity was measured with International Physical Activity Questionnaire. Descriptive statistics and multiple regression were performed. Results: Participants were ninety-nine Chinese older immigrants (mean age: 60.69 ± 7.62 years). Participants who had more children (p < .05), poor health status (p < .01), poor quality of life (p < .01), less social support (p < .01), and need help with activities of daily living (p < .05) had more depressive symptoms. Cognitive function, sleep quality, and physical activity were significantly associated with depressive symptoms. Conclusions & Implications: Poor cognitive function, poor sleep quality, and less physical activity were associated with depressive symptoms. Our results provide knowledge for developing culturally tailored self-management interventions for older Chinese immigrants with depressive disorder in managed care settings.

